# Renal Development and Blood Pressure in Offspring from Dams Submitted to High-Sodium Intake during Pregnancy and Lactation

**DOI:** 10.1155/2012/919128

**Published:** 2012-07-05

**Authors:** Terezila M. Coimbra, Heloísa D. C. Francescato, Ana Paula C. Balbi, Evelyn C. S. Marin, Roberto S. Costa

**Affiliations:** ^1^Department of Physiology, Faculty of Medicine, University of Sao Paulo, Avenida dos Bandeirantes 3900, 14049-900, Ribeirao Preto, SP, Brazil; ^2^Department of Pathology, Faculty of Medicine, University of Sao Paulo, Avenida dos Bandeirantes 3900, 14049-900, Ribeirao Preto, SP, Brazil

## Abstract

Exposure to an adverse environment in utero appears to programme physiology and metabolism permanently, with long-term consequences for health of the fetus or offspring. It was observed that the offspring from dams submitted to high-sodium intake during pregnancy present disturbances in renal development and in blood pressure. These alterations were associated with lower plasma levels of angiotensin II (AII) and changes in renal AII receptor I (AT_1_) and mitogen-activated protein kinase (MAPK) expressions during post natal kidney development. Clinical and experimental evidence show that the renin-angiotensin system (RAS) participates in renal development. Many effects of AII are mediated through MAPK pathways. Extracellular signal-regulated protein kinases (ERKs) play a pivotal role in cellular proliferation and differentiation. In conclusion, high-sodium intake during pregnancy and lactation can provoke disturbances in renal development in offspring leading to functional and structural alterations that persist in adult life. These changes can be related at least in part with the decrease in RAS activity considering that this system has an important role in renal development.

## 1. Introduction

The risk of hypertension, renal, and cardiovascular disease are in part determined before birth by intrauterine factors. Exposure to an adverse environment in utero appears to programme physiology and metabolism permanently, with long-term consequences for health of the fetus or offspring [1, 2]. The nephrogenesis in rats begins on embryonic day 12 and is completed at between 10 and 15 days after birth [3, 4]. Clinical and experimental evidence show that the renin-angiotensinn system (RAS) participates in renal development [5–11]. In the rat kidney, angiotensinogen expression is higher during the late gestation and newborn period whilst renin mRNA is detected from embryonic day 17 and is higher on embryonic day 20 and in newborns compared to adults [7, 8]. Renal AII content is several fold higher in newborn rats and mice than in their adult counterparts. The AII receptors are also expressed to a greater degree in newborn rats [9]. The mRNA for the type 1 AII receptor (AT_1_) has been detected in the renal glomeruli of newborn rats during cellular proliferation and differentiation [10]. 

Increase in fibronectin, *α*-SM-actin (*α*-smooth muscle actin), PCNA (proliferating cell nuclear antigen) and p-ERK (extracellular signal-regulated protein kinase) expressions were observed in the renal cortex of 1- and 7-day-old rats, followed by a decrease during renal development [12]. The activation of these proteins may be at least in part due to an increase in renal AII and MAPKs contents in renal cortex. Fibronectin is one of the most important components of the extracellular matrix (ECM). Formation of the ECM represents a key event in kidney cell differentiation [13, 14]. Fibronectin is a glycoprotein that can interact with some proteins from cell or ECM, inducing changes in cellular migration and adhesion [14]. It has also already been shown that human fetal mesangial cells express *α*-SM-actin [15, 16] and that during kidney development, a large-scale cell proliferation has been observed to occur [17]. 

Mutations in genes that encode components of the RAS are associated with autosomal recessive renal tubular dysgenesis [18]. Kidneys from fetus with tubular dysgenesis show lack of tubule differentiation, and marked thickening of arterial walls. These fetuses also present anuria, oligohydramnios, and skull ossification defects. The kidney of 3-week-old angiotensin-deficient mice showed pelvic dilation with atrophic papilla, medial hyperplasia of interlobular artery and afferent arterioles, glomerulus with axial mesangial expansion, decrease in glomerular area, patchy fibrosis, mononuclear infiltrate in the interstitium, and dilated tubules in the outer medulla with interstitial fibrosis around [19]. 

**Figure 1:**
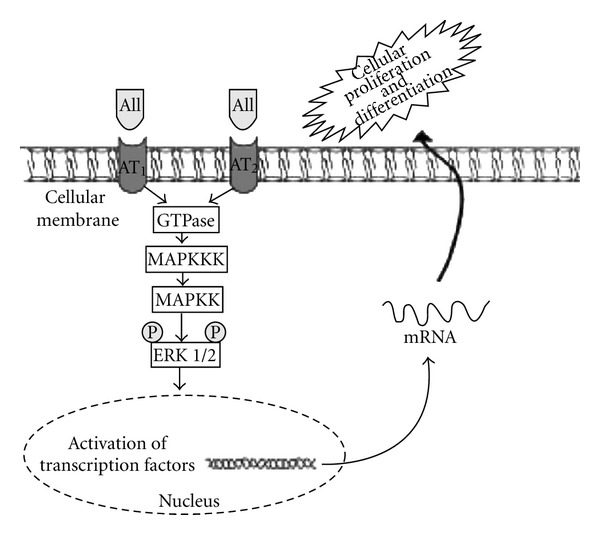
Schematic representation of angiotensin II (AII)-induced extracellular signal-regulated protein kinase (ERK) activation. AII acts through the type 1 AII (AT_1_) and type 2 AII (AT_2_) membrane receptors initiating intracellular signaling, with MAPK phosphorylation and activation of transcription factors in the nucleus, resulting in mRNA production for mediators of cellular differentiation and proliferation. GTPase: guanosine triphosphatase; MAPKKK: MAPKK kinase; MAPKK: MAPK kinase; ERK: extracellular signal-regulated protein kinase.

Many effects of AII are mediated through mitogen-activated protein kinase (MAPK) pathways [20–22]. The MAPK family is comprised of serine/threonine kinases, which are divided into three major subgroups: extracellular signal-regulated protein kinases (ERKs), c-Jun N-terminal kinases (JNKs)/stress-activated protein kinases, and p38 [23]. ERKs play a pivotal role in cellular proliferation and differentiation (Figure 1). In contrast, p38 and JNK MAPKs have been implicated in growth inhibition and apoptosis [22]. AII-converting enzyme modulates the expression of components of the MAPKs family in kidney from neonatal rats [21]. A decrease in glomeruli number was observed in 15-day-old embryos cultured for 48 and 120 hours with ERK or p38 inhibitors [23]. Choi et al. found that the treatment of newborn rats with enalapril for 7 days increases JNK 2 and p38 MAPKs expressions in the kidney, and that these increases were temporally associated with higher number of apoptotic cells [21].

Twenty-four-hour urine volume and liquid intake were higher in the pregnant females submitted to high-salt intake compared to the pregnant females from the control group. The body weight variation was also higher in the pregnant females from the experimental group compared to pregnant females from the control group [24]. Taken together, these results may reflect an increase of extracellular fluid volume in dams from the experimental group that can lead to a decrease of the RAS activity. The alteration in RAS activity can be also happening in the newborn rats. Since AII has an important role in kidney development, the lower AII levels in offspring in response to a high-salt intake of dams during gestation and lactation can provoke disturbances in renal function and structure in these animals. 

## 2. Renal Development in Offspring from Dams Submitted to High-Sodium Intake during Pregnancy

**Figure 2:**
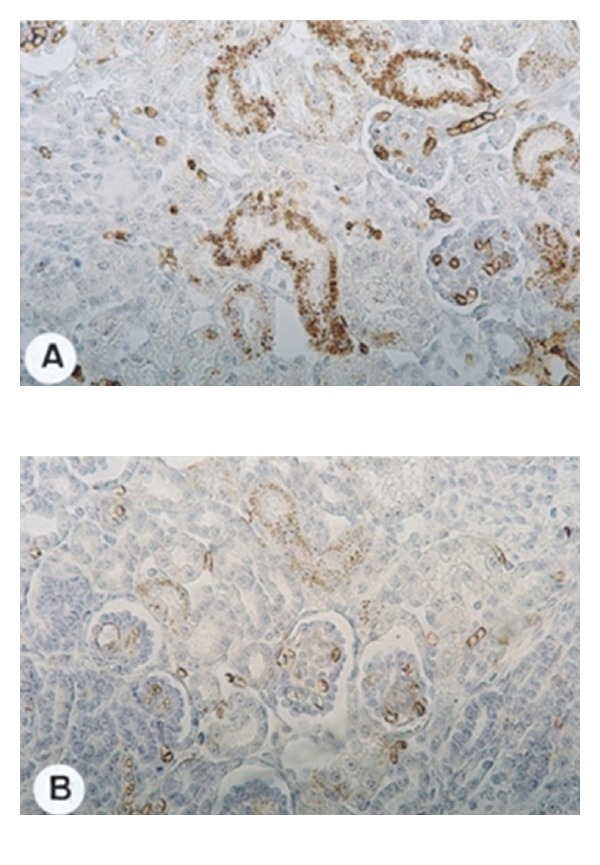
Immunolocalization of phospho-extracellular signal-regulated protein kinase (p-ERK) in renal cortex of offspring from dams submitted (B) or not (A) to high-sodium intake during pregnancy and lactation at 1 day of age (original magnification 280x). Note that the reactions are more intense in (A) than in (B).

Maternal high-sodium intake has been associated with kidney fetal development through its effects on plasma volume and on SRA system leading to changes in renal function and structure [12, 25, 26]. We observed, in previous studies, decrease in fibronectin, *α*-SM-actin, PCNA, p-ERK MAPK (Figure 2), AT_1_, and AII expressions in the renal cortex of 1-day-old pups from dams subjected to sodium intake compared to same age control rats [12]. Therefore, there was a temporal association between the reduction of AII and p-ERK MAPK expressions and the decrease of *α*-SM-actin, fibronectin, and PCNA expressions in renal cortex, suggesting a possible relationship between these findings. We also observed a decrease in the expression of AT_1_ receptors in 1-day-old rats born to mothers that received a higher-salt intake compared with controls of same age. Since kidney development is influenced by the RAS, possible functional changes in the RAS may occur in offspring due to the lower AII levels in response to a high-salt intake of dams during pregnancy. Some effects of AII are mediated through p-ERK pathway [20–22]. ERKs play a pivotal role in cellular proliferation and differentiation (Figure 1). On the other hand, increase in p-p38 and p-JNK MAPKs expressions were observed in 30-day-old pups of dams fed high-sodium diet. JNKs and p-38 have been implicated in growth inhibition and apoptosis.

Our results show that mesangial cell and interstitial fibroblast activation occurs during kidney differentiation, as confirmed by the increased expression of fibronectin and PCNA in the renal cortex from 1- and 7-day-old animals. The activation of these cells may be due, at least in part, to an increase in the renal AII production. Rats born from mothers that received a high-salt intake presented at 1st day of age a decrease in *α*-SM-actin, fibronectin, and PCNA expressions in the renal cortex, as well as a reduction in the number of AII-positive cells in glomerular and interstitial compartments compared with controls of the same age. There is some experimental evidence suggesting that AII can induce this phenotypic modification of these cells [27, 28]. The upregulation of *α*-SM-actin synthesis by these cells is frequently associated with increased cell proliferation and ECM. During kidney development, a large-scale proliferation has been observed [17]. Therefore, changes in the renal expression of AII in animals born from mothers fed high-salt diet might affect renal cell proliferation and ECM production during postnatal development. A decline in GFR was also observed in 30-day-old animals from this group. These data demonstrate that disturbances occurring during intrauterine life can induce persistent alterations in the biology of the pups in adult life.

Koleganova et al. [26] verified that maternal high-salt intake leads to reduced final number of glomeruli, glomerulomegaly, and albuminuria in the offspring. Cardoso et al. [25] found that prenatally and postnatally sodium-overloaded rats showed increased proteinuria and reduced glomerular filtration rate (GFR) that were associated with kidney oxidative stress. Therefore, this damage in the offspring renal tissue may be related at least in part with disturbances in antioxidant defense in dams. Besides this it, was observed that the antioxidant defenses were altered in newborns [29]. Beauséjour et al. [30] showed that the marker of oxidative stress (nitrotyrosine expression), the producer of nitric oxide (nitric oxide synthases), and apoptotic index were increased in kidneys from pregnant rats that consumed a diet with high sodium which was associated with decrease of Na^+^-K^+^-ATPase mRNA and activity. In a study performed with the renal proximal tubular cells, it was found that nitric oxide-derived products (such as peroxynitrite) inhibited Na^+^-K^+^-ATPase activity via the oxidation of thiol groups of this enzyme [31]. Guzman et al. reported that this inhibition was accompanied by a reduction in Na^+^-dependent solute transport [32]. In addition, it was also observed that endogenous nitric oxide plays a direct inhibitory role in Na^+^-K^+^-ATPase activity in the kidney [33]. Koleganova et al. (2011) showed that the expression of *α*_1_- Na^+^-K^+^-ATPase protein and RNA levels at term was significantly lower in offspring of dams with high sodium diet [26].

**Figure 3:**
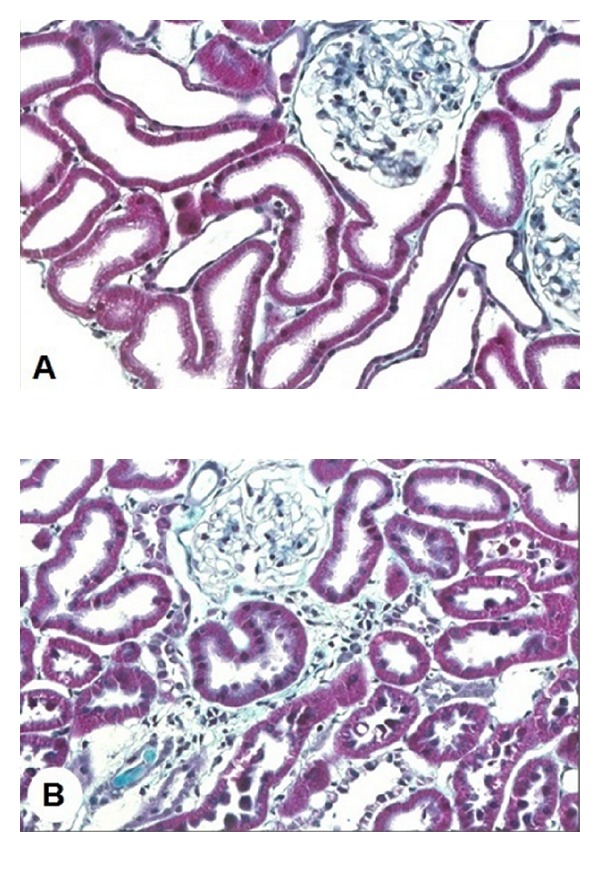
Masson's trichrome stained histological sections of renal cortex of rats from dams submitted to high-sodium intake (B) and of the same age control rats (A) at 120 days of age (original magnification 280x).

We also found [24] higher scores for glomerulosclerosis and tubulointerstitial lesions in renal cortex of 120-day-old offspring from dams subjected to increase in saline intake compared to the same age control rats (Figure 3). These alterations were associated to an increase in the number of macrophages in renal cortex and glomeruli. Macrophages activated by hypoxia and the oxidative stress present in the injured tissue can release fibrogenic peptides, such as TGF-*β* (transforming growth factor *β*), interleukin-1, endothelin, AII, and radical oxygen species, contributing to intensify the inflammatory process and fibrosis [34].

## 3. Blood Pressure in Offspring from Dams Submitted to High-Sodium Intake during Pregnancy

Higher blood pressure was observed in newborns from dams submitted to high-sodium intake during pregnancy and lactation [12, 24, 25, 35]. It was observed by da Silva et al. that adult rats submitted to prenatal salt overload presented RAS functional disturbances [36]. They found that plasma renin activity did not modify in response to high-salt intake in the adult rats submitted to a high-salt environment during the prenatal period. There is evidence that AII receptors in brain regions involved in cardiovascular control and water and electrolyte balance are influenced by perinatal high-sodium intake [37, 38]. It was also shown that pressor response to peripheral AII is increased in adult rats submitted to perinatal salt ingestion compared to basal salt intake [38, 39]. However, study from Porter et al. [40] showed that limiting maternal exposure to a high-sodium diet to the prenatal period is not sufficient to program lasting hypertension in offspring, but an enhanced pressor and tachycardic response to acute stress does persist in adulthood. Therefore, the authors concluded that repeated enhanced response to acute stress observed in these animals has the potential to produce permanent cardiovascular disease.

## 4. Conclusion

High-sodium intake during pregnancy and lactation can provoke disturbances in renal development in offspring leading to functional (proteinuria, decrease in GFR, and higher blood pressure) and structural alterations (lower glomeruli numbers, glomerulomegaly, glomerulosclerosis, and tubulointerstitial lesions) that persist in adult life. These changes can be related at least in part with RAS activity that has an important role in renal development.
